# TSPYL2 Is Important for G1 Checkpoint Maintenance upon DNA Damage

**DOI:** 10.1371/journal.pone.0021602

**Published:** 2011-06-27

**Authors:** Kin Pong Tao, Sze Wan Fong, Zhihong Lu, Yick Pang Ching, Kin Wang Chan, Siu Yuen Chan

**Affiliations:** 1 Department of Paediatrics and Adolescent Medicine, Center of Reproduction, Development and Growth, Li Ka Shing Faculty of Medicine, The University of Hong Kong, Hong Kong, China; 2 Department of Anatomy, Li Ka Shing Faculty of Medicine, The University of Hong Kong, Hong Kong, China; University Medical Center Hamburg-Eppendorf, Germany

## Abstract

Nucleosome assembly proteins play important roles in chromatin remodeling, which determines gene expression, cell proliferation and terminal differentiation. Testis specific protein, Y-encoded-like 2 (TSPYL2) is a nucleosome assembly protein expressed in neuronal precursors and mature neurons. Previous studies have shown that TSPYL2 binds cyclin B and inhibits cell proliferation in cultured cells suggesting a role in cell cycle regulation. To investigate the physiological significance of TSPYL2 in the control of cell cycle, we generated mice with targeted disruption of *Tspyl2*. These mutant mice appear grossly normal, have normal life span and do not exhibit increased tumor incidence. To define the role of TSPYL2 in DNA repair, checkpoint arrest and apoptosis, primary embryonic fibroblasts and thymocytes from *Tspyl2* deficient mice were isolated and examined under unperturbed and stressed conditions. We show that mutant fibroblasts are impaired in G1 arrest under the situation of DNA damage induced by gamma irradiation. This is mainly attributed to the defective activation of *p21* transcription despite proper p53 protein accumulation, suggesting that TSPYL2 is additionally required for *p21* induction. TSPYL2 serves a biological role in maintaining the G1 checkpoint under stress condition.

## Introduction

Nucleosome assembly proteins (NAPs) play important roles in the control of cell proliferation. They are divided into NAP, SET and TSPY families according to the amino acid conservation of the NAP domain. NAPs are involved in cell cycle control through regulating the transcription of cell cycle genes and through interaction with cyclins [1], [2]. Knockout of NAP1, the prototypic NAP family member, is embryonic lethal in *Drosophila* [3]. In yeast, NAP1 binds the mitotic cyclin Clb2 (homologue of cyclin B) and the protein kinase Gin4, thereby inducing cell cycle events [4], [5]. Testis-specific protein, Y-encoded (TSPY) also interacts with cyclin B and enhances cyclin B-CDK1 phosphorylation [6]. As the first TSPY family member being identified, TSPY accelerates cell proliferation, and is related to testicular and prostate cancers [7], [8], [9].

TSPY-like 2 (TSPYL2, also named as CDA1, CINAP, DENTT, NP79, Se20-4 and TSPX) is involved in cell cycle control, but in an opposite manner to the effect of TSPY. Whereas TSPY increases cyclin B-CDK1 activity, TSPYL2 inhibits its activity. The NAP domain performs the same function of binding cyclin B, but the acidic C-terminal tail in TSPYL2, and in another NAP member SET, is responsible for the inhibitory effect on cyclin B-CDK activity [6]. In agreement to this finding, SET overexpression retards cell cycle transition at G2/M [10]. By contrast the effect of TSPYL2 on cell cycle profile is not obvious although it can also arrest cell growth and inhibit DNA synthesis [11]. TSPYL2 may play a role in arresting cell growth upon DNA damage, as it can be induced by camptothecin, a topoisomerase inhibitor that induces DNA strand breaks [12]. TSPYL2 is also upregulated by TGFβ1, a cytokine that blocks the advancement from G1 to S phase, in responsive non-small-cell lung carcinoma cell lines [13]. Together with the fact that TSPYL2 can repress cyclin B-CDK1 activities, the major regulator complex during G2 to M phase progression, the data suggest that TSPYL2 can inhibit both G1/S and G2/M phases of the cell cycle. In agreement with the notion that TSPYL2 is a negative cell cycle regulator, TSPYL2 is silenced in glioma tissues, malignant lung tissues and certain lung tumor cell lines [14], [15]. Overexpression of TSPYL2 in human lung and breast cancer cell lines results in reduced growth potential [15]. Nevertheless, direct evidence of a physiological function of TSPYL2 in cell cycle control is lacking. Loss-of-function analysis is a powerful approach to address this question.

Loss-of-function of NAPs has been associated with human diseases. A frameshift mutation which results in truncation of the NAP domain in TSPYL1 is found in sudden infant death with dysgenesis of the testis syndrome [16]. Mouse embryos with deletion of the *Nap1l2* gene exhibit neural tube defects closely resembling spina bifida and anencephaly, and NAP1L2 may be related to X-linked neural tube defects in humans [17], [18]. It is essential to validate the *in vitro* findings of TSPYL2 on cell cycle control and tumor suppression *in vivo*, as exemplified by elegant studies on various mouse mutants of p53 which demonstrate that *in vivo* findings can contradict the results in biochemical assays and cell line studies [19]. Here we report the generation and analysis of TSPYL2 deficient mice. The mutant mice generated are viable, fertile and do not exhibit increased tumor incidence. Ionizing radiation (IR) was employed for studying the DNA damage response without the complications of stalled transcription from ultraviolet light and topoisomerase inhibitors [20]. Primary cultures of mouse embryonic fibroblasts (MEFs) and thymocytes were isolated as they serve as well-established models for studying cell cycle control and apoptosis, respectively. We find that MEFs lacking functional *Tspyl2* proliferate normally, but are impaired in G1 arrest following DNA damage. Despite proper stabilization of p53, induction of *p21* transcription is specifically impaired. Our data demonstrate that loss of TSPYL2 function is not deleterious to the development of mice, and reveal the importance of TSPYL2 in the maintenance of G1 checkpoint under stress condition.

## Materials and Methods

### Ethics statement

Mouse experiments were approved and performed according to the instructions of Committee on the Use of Live Animals in Teaching and Research at the University of Hong Kong (approval ID: CULATR 1643-08).

### Gene targeting, genotyping and mouse lines

In view of the potential importance of TSPYL2 in cell proliferation and brain development, a conditional knockout construct was designed. The neomycin resistance (neo) cassette with the phosphoglycerate kinase 1 promoter and polyA was franked by frt sites and inserted into intron 5 of *Tspyl2*. This allowed the removal of the neo cassette by flip recombinase so that it would not interfere with the transcription of *Tspyl2*. Exons 2 to 5, together with the neo cassette oriented in opposite direction to *Tspyl2* transcription, was flanked by loxP sites. Exons 2 to 5, which encoded the NAP domain, could be conditionally deleted by expression of Cre recombinase. The targeting vector contained 4.0 kb of 5′ and 1.9 kb of 3′ homology arms. Embryonic stem (ES) cells were derived in house from 129Sv/Ev embryos by the Transgenic Core Facility at HKU, which also provided the service for electroporations and blastocyst injections. Homologous recombination in the G418 resistant ES clones was detected by Southern blot analysis using standard procedures. Probes for Southern blotting were derived from PCR, cloned and sequence verified. Chimeric mice were generated by injecting the targeted ES clones into C57BL/6 blastocysts, and mated to 129Sv/Ev mice. Germline transmission of the mutant *Tspyl2* allele (*Tspyl2^m^*) was validated by PCR genotyping using primers binding *Tspyl2* exon 5 (F5: 5′ CTACTATATGAGACGAGG) and *neo* (neo-R: 5′ TGAAGAACGAGATCAGCAGC). The wildtype *Tspyl2* allele was detected using primers for exon 5 (F5) and exon 6 (R6: 5′ TGGTCAGGATCTTCACTGTC). The mouse line was called Tspyl2^m^ and maintained in pure 129Sv/Ev background.

To remove exons 2 to 5 in *Tspyl2* ^m^ allele for the generation of Tspyl2 deleted mouse line, *Tspyl2* ^m/Y^ mice were mated to female transgenic mice that expressed Cre recombinase under the promoter for *zona pellucida glycoprotein 3* [21]. The transgenic females originated from injected FVB/N oocytes and were subsequently bred into Swiss outbred background. Germline Cre-mediated excision of exons 2 to 5 was confirmed by PCR using primers for intron 1 (C1: 5′ CTTAGCATCTAGACCTACAGC) and exon 6 (R6). First generation offspring with the deleted allele (*Tspyl2* ^−^) but not the *Cre* transgene as determined by PCR were used to establish the Tspyl2^−^ line by brother to sister mating.

### Northern Blot

Ten micrograms of RNA were resolved in 1% formaldehyde agarose gel. The blot was hybridized in Church buffer at 65°C with [αP^32^]-dCTP labeled probes covering nucleotide 545 - 1033 of *Tspyl2* mRNA (NM_029836.3). The blot was washed twice with 2X SSC/0.1% SDS at 65°C for 10 minutes each, and then three times with 1X SSC/0.1% SDS at 65°C for 15 minutes each.

### Brain histology and immunohistochemistry

Mice were intraperitoneally injected with 95 µg/g 5-bromo-2-deoxyuridine (BrdU) in saline for six consecutive days. Brains were collected after transcardial perfusion with 4% paraformaldehyde and fixed overnight. Brains were cryoprotected in 30% sucrose, and serial coronal sections of 40 µm were cut using a freezing microtome. After mounting, sections were incubated in citrate buffer (pH 6.0) at 85°C for 25 minutes. After 3 washes in PBS, sections were incubated with 37°C prewarmed 2M hydrochloric acid for 10 minutes, followed by 0.1 M borate buffer (pH 8.5) for 15 minutes for partial denaturation of DNA before commencing to conventional immnohistochemistry protocol. Staining was performed with anti-BrdU (Developmental Studies Hybridoma Bank) and Alexa Fluor® 488 goat anti-mouse IgG antibody (Invitrogen). The epiflorescence was observed using Axioplan-2 Carl Zeiss system, and the number of BrdU positive cells in the whole dentate gyrus was counted on one-in-six sections, omitting the outermost focal plane. The total number of BrdU positive cells being counted was multiplied by six to obtain the number for the entire dentate gyrus. For the lateral ventricle, the number of BrdU positive cells in corresponding region (Bregma level of −0.8 mm) was counted in one section, and the average was taken from three mice for each genotype.

### Cell harvest, culture and treatments

MEFs were isolated from 12.5–14.5 day post-coitum embryos from heterozygous females mated with mutant males, and routinely passaged using the standard 3T3 protocol. MEFs were cultured in Dulbecco's modified Eagle's medium (Invitrogen) supplied with 10% fetal bovine serum (HyClone) at 37°C and 5% CO_2_. Single cell suspension of thymocytes was obtained by passing the dissected thymus from 2-months old mice through a 40 µm cell strainer (Falcon) followed by red blood cell lysis. Thymocytes were cultured overnight in Roswell Park Memorial Institute medium (Invitrogen) supplemented with 10% fetal bovine serum. For synchronization of MEFs in G1 or G2 phase, cells were incubated with 5 µg/ml aphidicolin (Biomol) or 80 ng/ml nocodazole (Sigma-Aldrich) for 12 hours. The IR experiments were performed by Gammacell 3000 Elan irradiator (MDS Nordion).

### Flow cytometry

For cell cycle analysis, culture cells were collected by trypsinization, and fixed overnight at −20°C in 70% ethanol. Right before Flow analysis, cells were suspended in propidium iodide (PI) staining solution (Invitrogen) containing RNase for 15 minutes at room temperature. Cells were applied to EPICS Elite Flow Cytometer (Beckman Coulter) or LSRII (BD Biosciences). Cell cycle profiles were analyzed by ModFit® (Verity Software House). For analysis of apoptotic response, freshly isolated thymocytes from mice untreated or irradiated at 4 Gy were washed and stained with FITC tagged anti-CD4 and PE-Cy5 tagged anti-CD8 antibodies (BD Biosciences) for 1 hour, followed by Flow analysis. For Annexin V-PI (AVPI) experiment, thymocytes were collected from untreated mice, cultured overnight and subjected to 10 Gy IR. Cultures were collected at various time points for staining with BD Pharmingen™ Annexin V: FITC Apoptosis Detection Kit II according to manufacturer's instruction. Viability and apoptotic proportions were analyzed after collecting >20,000 cells with proper gating.

### Immunocytochemistry and western blot

For immunocytochemistry, anti γ-H2AX (Upstate Biotech) and AlexaFlor® 594 anti-mouse IgG (Invitrogen) were used as primary and secondary antibodies. The primary antibodies used in western analysis included p53 (sc1311, Santa Cruz), acetyl-Lys379 p53 (#2570, Cell Signaling) and p21 (sc6246, Santa Cruz). Thirty µg of protein samples were loaded in parallel gels, and anti-actin (Sigma) was used as loading control. HRP-tagged secondary antibodies were detected using ECL Plus system (Amersham).

### RT-PCR and real-time quantitative RT-PCR (qPCR)

RNA was isolated by Trizol (Invitrogen). After digestion with DNase I, 1 µg of RNA was reverse transcribed with SuperScript II (Invitrogen). One-tenth of the resulting cDNA sample was used for PCR. Semi-quantitative PCR was performed at 95°C for 5 min, followed by 30 cycles of denaturation at 95°C for 45 seconds, annealing at 58°C for 30 seconds, and elongation at 72°C for 45 seconds. The final extension was 7 minutes. The primers used were listed as follows (5′ to 3′): F1: GGTTGCAGAGCCCAGCAG; R2: CACATTGGTTGATCAAGATTG; F2: GAGACCTCATCATCCAGCAT; R5: CCTCGTCTCATATAGTAG; F5; R6; Hprt-F: AACTGGAAAGAATGTCTTGATTG; Hprt-R: TCAAATCCAACAAAGTCTGGC; Tspyl1-F: ATGGAAACAGCTGAGCCCTT; Tspyl1-R: AAAGGCAGGCGGATTTCTGA; Tspyl2-QF: GGTCAAAGCATTCCTCAACC; Tspyl2-QR: GGTCCTGTACCTGAAGATTG; Tspyl3-F: GCGAGGCTTAGAATATCCAG; Tspyl3-R: AGGTGCAAAGCTACCTTGCA; Tspyl4-F: AGCTTCTCCAAAGCTCGTAG; Tspyl4-R: TGAGTGTTCCCAGGAGTCTT; Tspyl5-F: TTTGGGAGACTGGGGCATTG; Tspyl5-R: CAGAACACAGCAACCCTAAC. qPCR was performed using the iQ SYBR Green Supermix with ROX (Bio-Rad) on 7900HT System (Applied Biosystems) according to manufacturers' instruction. The expression of genes was calculated using the ΔΔCT method after normalization by *Hprt* (hypoxanthine guanine phosphoribosyl transferase), which showed a similar level of expression in all samples tested. Primers used were: Hprt-F, Hprt-R, Tspyl2-QF, Tspyl2-QR, p21-F: CCTGGTGATGTCCGACCTGTT; p21-R: GGGGAATCTTCAGGCCGCTC; Mdm2-F: TGAGACAGAAGAGAACACAG; Mdm2-R: CTTCCAATAGTCAGCTAAGG; Noxa-F: TGCCAGCAGACTTGAAGGAC; Noxa-R: GGAACCAAAAGCAAGCGAGC; 14-3-3σ-F: TGTCTGTCCATCCTCGCAGT; 14-3-3σ-R: CCTCCTCGTTGCTCTTCTGC; Bax-F: CTCAAGGCCCTGTGCACTAA; Bax-R: ATGTGGGGGTCCCGAAGTAG.

### Statistical analysis

Student t-test was used for pairwise comparison, and two-way ANOVA followed by Bonferroni post-test were used for time-point qPCR analysis. All graphs were presented as the average ± SEM.

## Results

### Generation of Tspyl2 knockout mice

**Figure 1:**
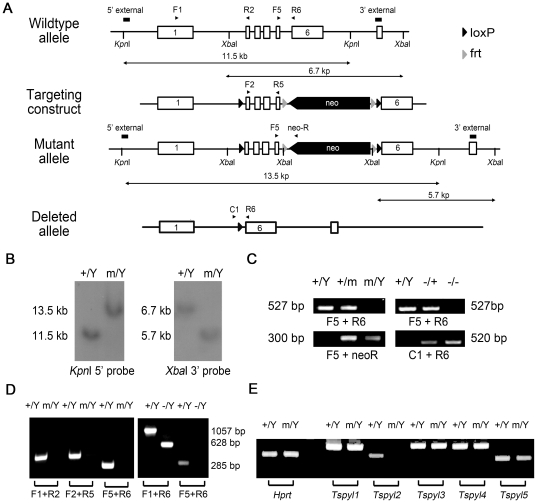
Generation of *Tspyl2* knockout mice. (**A**) Strategy for the target inactivation of *Tspyl2*. The targeting vector was constructed with *neo* expression cassette inserted into intron 5 of *Tspyl2* in reverse orientation. The deleted allele was generated through Cre-mediated excision. Exons 1 and 6 were numbered, and the positions of external probes for Southern Blot and primers for PCR were marked. (**B**) Southern blot analysis using the 5′ and 3′ probes on *Kpn*I and *Xba*I digested ES cell DNA showed expected fragment sizes. +/Y: wildtype; m/Y targeted mutant. (**C**) PCR genotyping of mice with PCR primers indicated. Typical results with DNA from wildtype male (+/Y), heterozygous *Tspyl2* ^+/m^ female (+/m), *Tspyl2* ^m/Y^ male (m/Y), *Tspyl2* ^+/−^ female (+/−), *Tspyl2* ^−/−^ female (−/−). (**D**) Left: RT-PCR with 35 cycles showed that insertion of *neo* eliminated the normal *Tspyl2* transcript in *Tspyl2^m/Y^* brain; Right: RT-PCR with 30 cycles showed the correct deletion of *Tspyl2* exons in *Tspyl2^−/Y^*. Primers were numbered according to exon binding sites. (**E**) RT-PCR indicated that m/Y brain had no upregulation of other family members of *Tspyl* genes.

*Tspyl2* deficient mice were generated using the targeting strategy shown in Fig. 1A. Two targeted clones were obtained after screening around one thousand ES colonies. The disruption of *Tspyl2* was confirmed by Southern blot analysis with both 5′ and 3′ external probes (Fig. 1B). Chimeric mice were generated from blastocyst injection of targeted clones and crossed with wildtype 129Sv/Ev mice to generate the Tspyl2^m^ line of pure genetic background. Genotypes of the pups were identified by PCR (Fig. 1C). Mice of various genotypes occurred in the correct Mendelian ratio. The *Tspyl2* deleted line (Tspyl2 ^−^) was generated by crossing the Tspyl2^m^ line to outbred transgenic females expressing *Cre* and genotyped by PCR (Fig. 1C).

To characterize the Tspyl2^m^ line, brain RNA samples were collected for analysis. RT-PCR with primers amplifying exons 1 to 2, 2 to 5 and 5 to 6 under extended cycle number of 35 revealed that the normal *Tspyl2* transcript was eliminated in the *Tspyl2* ^m/Y^ mice. By contrast, the exon 2 to 5-deleted transcript was detected after 30 PCR cycles in Tspyl2^−/Y^ mice (Fig. 1D). The data indicated that none of the previously reported transcript variants of mouse *Tspyl2* was detectable in our two mouse lines [22]. To assess the potential compensatory effect from other TSPYL homologues, semi-quantitative RT-PCR was performed and no obvious upregulation of other TSPY family members was detected (Fig. 1E). In summary, the insertion of *neo* results in no stable *Tspyl2* transcript being detected. Tspyl2^m^ line was effectively a Tspyl2 knockout line. This agreed with the finding that homozygous and hemizygous mice in both Tspyl2^m^ and Tspyl2 ^−^ lines were phenotypically normal. We performed our analysis on the Tspyl2^m^ mice since they were in pure 129/SvEv genetic background.

### Normal proliferation of neuronal precursor cells in Tspyl2^m^ animals

**Figure 2:**
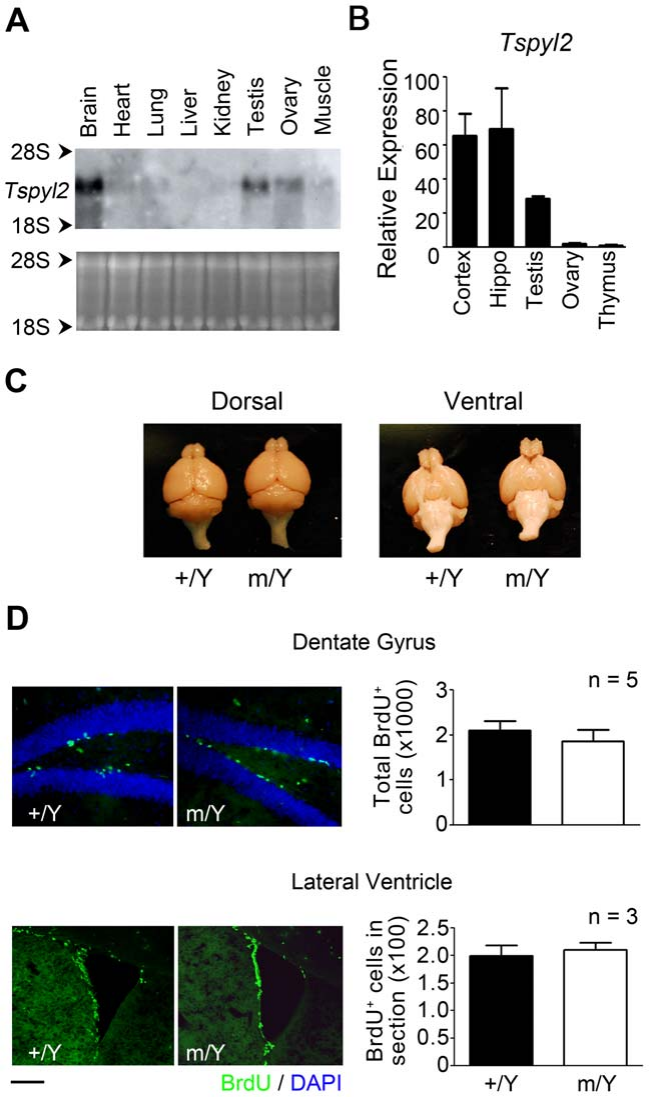
*Tspyl2* mutant mice have normal proliferation of neuronal precursor cells. (**A**) The expression pattern of *Tspyl2* as revealed by northern blot. Equal loading of RNA was visualized by ethidium bromide staining. (**B**) qPCR analysis of *Tspyl2* transcript in cerebral cortex, hippocampus (Hippo), testis, ovary and thymus in adult mice (n = 3–5). The relative expression of each tissue is expressed as the fold difference when compared with the thymus after normalization with *Hprt*. (**C**) Gross morphology of the brain dissected from wildtype (+/Y) and *Tspyl2* ^m/Y^ (m/Y) adult mice after perfusion fixation. (**D**) BrdU staining in dentate gyrus of hippocampus and lateral ventricle of 2 months old adult mice. Nuclei were stained by DAPI. Scale bar: 50 µm. The number of BrdU-positive cells was counted and shown on the right. Error bar: SEM.

*TSPY* is functional in humans and rats but not in mice [23]. To detect any species difference in the expression of its X-chromosome homologue *TSPYL2*, we checked its expression in mice. We previously reported that *TSPYL2* is highly expressed in the human heart, brain and lung as revealed by Northern blotting [24]. High expression in the human heart was confirmed in two additional RNA samples (data not shown). In mice, high expression of *Tspyl2* was detected in the brain but not the heart, and lower expression was detected in gonads (Fig. 2A). *Tspyl2* expression in cerebral cortex, hippocampus, gonads and thymus was further compared by qPCR (Fig. 2B). Despite the expression of *Tspyl2* in gonads, *Tspyl2* ^m/m^ and *Tspyl2* ^m/Y^ mice were of normal fertility. The morphology and size of *Tspyl2* mutant brain were also comparable to that of wildtype littermates (Fig. 2C). Since it has been reported that TSPYL2 protein is detected in proliferating neuron precursors in the adult brain, we further investigated the consequence of gene knockout in the two brain areas where these cells are found [25]. The numbers of BrdU labeled neuronal precursors in dentate gyrus of the hippocampus and subependyma surrounding the lateral ventricles were similar between wildtype and mutant (Fig. 2D). The result shows that TSPYL2 is not essential for proliferation of neuron precursors in adults.

### Tspyl2 mutant cells proliferate normally in culture

**Figure 3:**
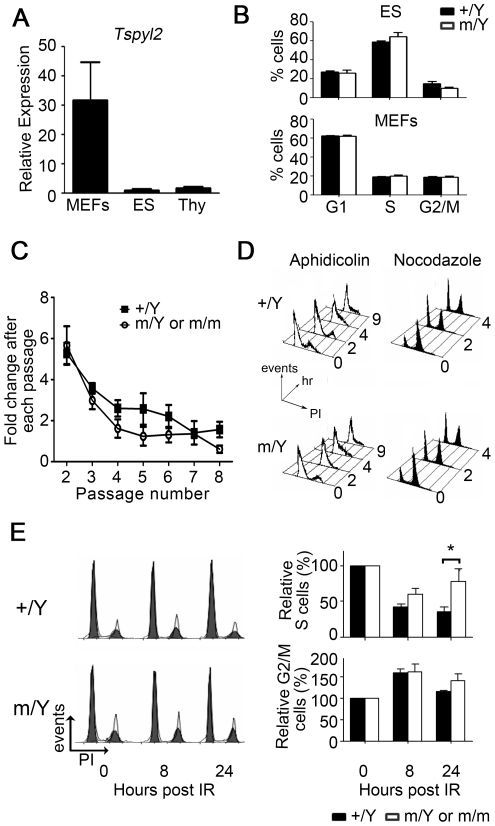
*Tspyl2* is dispensable for cell proliferation, but responsible for maintaining G1 checkpoint upon IR. (**A**) qPCR analysis of *Tspyl2* in MEFs, ES cells and thymocytes (Thy) (n = 3–4). The relative expression was expressed as the fold difference when compared with thymocytes after normalization with *Hprt*. (**B**) Rapidly growing ES cells and MEFs from wildtype (+/Y) and *Tspyl2* ^m/Y^ mutants (m/Y) were subjected to PI based cell cycle analysis. Cell populations from three independent experiments were summarized. Error bar: SEM. (**C**) MEFs harvested from wildtype (+/+ or +/Y, n = 4) and *Tspyl2^m^* mutants (m/Y or m/m, n = 5) were passaged under standard 3T3 protocol. Mutant and wildtype littermate MEFs proliferated at a similar rate after prolonged passaging. (**D**) MEFs were transiently treated with 5 µg/ml aphidicolin (unfilled) or 80 ng/ml nocodazole (black filled) for 12 hours for synchronization in G1 or G2 phase. Progression through cell cycle arrest after the release was measured by PI based Flow analysis. Numbers indicated on the right represent the hours released from synchronization treatment. (**E**) MEFs of passage 3 were exposed to 10 Gy IR and fixed at indicated time points for Flow analysis. Left: A representative result showing the cell cycle analysis using ModFit LT software. The calculated G1 and G2/M phases were represented by the two filled peaks, and the stripped portion in between corresponding to S phase. Right: The percentage of cells at S and G2/M from four independent experiments. Data were presented as mean ± SEM. A significantly higher percentage of mutant MEFs had entered S phase 24 hours after IR (*: p<0.05, n = 9 for each group).

We wondered whether TSPYL2 behaved as a tumor suppressor, but we observed no spontaneous tumor development in our mutant mice and their life span was normal. To reveal any subtle role of TSPYL2 in cell proliferation, we used ES cells, primary MEFs and thymocytes as sources of normal cells. The expression of *Tspyl2* in these cell types was measured by qPCR (Fig. 3A). The level of Tspyl2 expression in thymocytes was similar to that of thymus and set as 1 for comparison. ES cells and MEFs were subjected to PI based cell cycle analysis. As *Tspyl2* is subjected to X-inactivation, heterozygous MEFs were excluded from analysis as random inactivation of *Tspyl2* had occurred [26]. Using early passage cells, *Tspyl2* ^m/Y^ cells had a normal cell cycle profile, with no apoptotic or aneuploidy populations. The proportion of G1, S and G2/M cell populations was similar between wildtype and mutant from three independent experiments (Fig. 3B). In terms of senescence induced by culture stress, proliferation curves of wildtype and mutant MEFs were comparable and so did staining with senescence-associated β-galactosidase at passage 6 (Fig. 3C and data not shown). MEFs of both genotypes could be spontaneously immortalized. Next, early passage MEFs were synchronized with aphidicolin and nocodazole for transiently arresting cells in G1 and G2 phase, respectively. Re-entry into cell cycle through these checkpoints was measured by Flow cytometry (Fig. 3D). Again, *Tspyl2* mutant MEFs showed normal advancement of cell cycle through both checkpoints after drug release.

### Tspyl2 mutant MEFs are defective in cell cycle arrest upon IR

Previous studies have shown that the transcription of *TSPYL2* is activated upon treatment with camptothecin, a potent inducer of DNA strand breaks and inhibitor of topoisomerase I [12]. To further dissect the importance of *Tspyl2* in cell cycle control, we induced DNA damage by IR. As ES cells are atypical in their response to DNA damage, only MEFs were used in this study as sources of non-transformed cells [27]. Early passage MEFs from wildtype and mutant embryos were subjected to 10 Gy IR for the induction of DNA double strand breaks. The proportion of S phase cells was determined by Flow cytometry (Fig. 3E). With functional checkpoint activation, normal MEFs undergo cell cycle arrest in response to DNA damage for DNA repair. Initiation of G1 and G2 checkpoints in mutant MEFs was normal, as indicated by a similar reduction in the percentage of S phase cells and increase in G2/M cells in wildtype and mutant MEFs at 8 hours after IR. The proportion of S phase cells in the wildtype remained low 24 hours after IR (35.9% ±6% of untreated, n = 9). By contrast, a significantly larger proportion of mutant MEFs had returned to S phase (77.51% ±17.3% of untreated, n = 9, p<0.05). This suggests a defect in the maintenance instead of initiation of the G1 checkpoint in the mutants.

**Figure 4:**
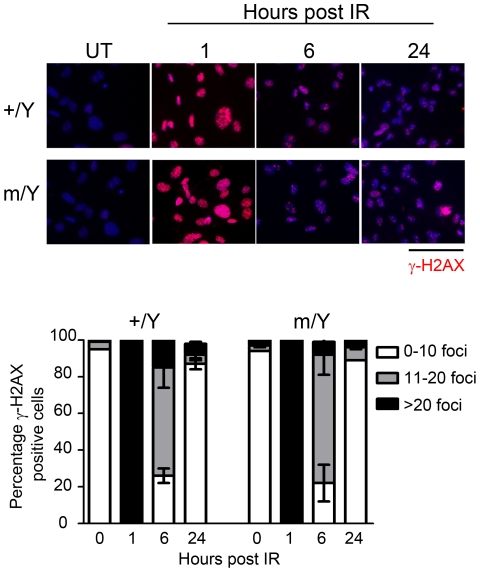
Normal DNA repair in *Tspyl2* mutant MEFs. MEFs from wildtype (+/Y) and *Tspyl2* ^m/Y^ mutant (m/Y) were irradiated at 10 Gy and collected at indicated time points for γ-H2AX immunocytochemistry for the detection of DNA damage foci. Scale bar: 200 µm. Cells (>200) with various number of lesion foci were counted and the degree of damage was summarized in the histogram on the bottom (n = 2 for each genotype). Data represented as mean value ± SEM from two independent experiments.

Upstream of cell cycle arrest, histone H2AX is rapidly phosphorylated on serine 139 (γ-H2AX) which serves as a label of DNA damage loci [28]. Mutant MEFs had a normal time course of DNA repair as reflected by γ-H2AX counting. One hour after IR, wildtype and mutant MEFs were both able to trigger the H2AX phosphorylation, indicating normal recognition of DNA lesion. After 6 and 24 hours, the degree of lesion gradually decreased which indicated DNA repair. The recovery rate of DNA damage in the mutant MEFs was comparable with that of the wildtype controls (Fig. 4).

### Impaired *p21* transcription upon IR in mutant MEFs

**Figure 5:**
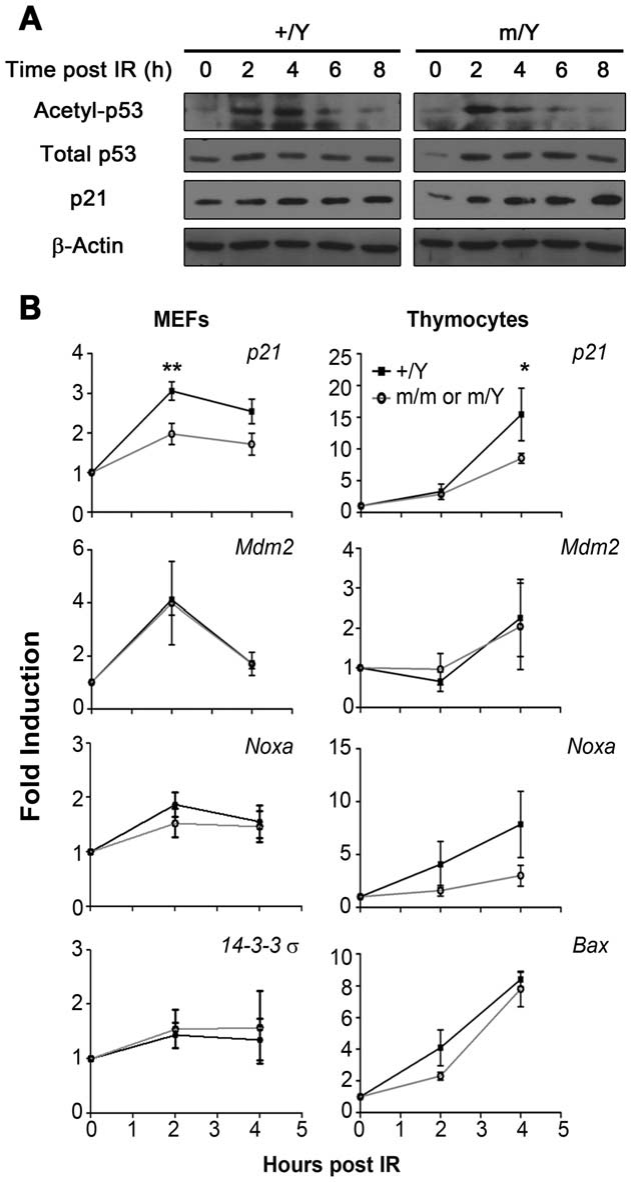
Impaired transcription of *p21* downstream of p53 stabilization upon IR in mutant cells. (**A**) Western Blot analysis of lysates from wildtype (+/Y) and *Tspyl2* ^m/Y^ mutant (m/Y) MEFs collected at indicated time points post 10 Gy IR to acetyl-p53 at Lys379, total p53 and p21. Actin served as a loading control. (**B**) qPCR analysis in MEFs (left panel) and thymocytes (right panel) after IR at 10 Gy. Transcription levels of indicated checkpoint genes were normalized by *Hprt*. The fold induction of individual gene is expressed as mean value ± SEM when compared to its untreated control (n = 5 per group). Data points showing asterisk indicate statistical significance between wildtype and mutants as revealed by Bonferroni post-test after 2-way ANOVA (*: p<0.05, **: p<0.01).

In the presence of DNA damage, there is a rapid accumulation of p53 and p21. In addition, acetylation of p53 promotes recruitment of coactivator such as CBP to the p21 promoter resulting in transcription activation [29]. While we observed no consistent difference in the basal level of p53 and p21 between wildtype and mutant MEFs, Lys379 of p53 was clearly acetylated upon IR. There was also an increase in the protein level of p53 and p21 after 10 Gy IR in both genotypes (Fig. 5A). As a member of NAPs, TSPYL2 is likely to regulate transcription of various checkpoint genes in DNA damage response. In support of this, *Tspyl2* mutant MEFs were defective in *p21* induction after exposure to 10 Gy IR (p<0.01). Thymocytes also showed impaired *p21* transcription activation 4 hours after IR (p<0.05, Fig. 5B). Interestingly, the activation of other p53 target genes *Mdm2*, *14-3-3σ* and the apoptotic genes *Noxa* and *Bax* was not significantly different between wildtype and mutant MEFs and thymocytes. The baseline level of expression of the above genes was similar between wildtype and mutant in the qPCR assay.

### Tspyl2 mutant thymocytes undergo apoptosis as control cells upon IR

**Figure 6:**
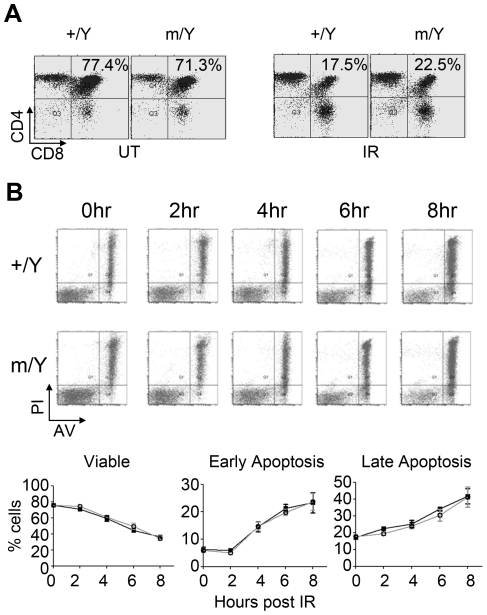
Normal apoptotic response of *Tspyl2* mutant thymocytes after IR. (**A**) Thymocytes isolated from untreated (UT) wildtype and *Tspyl2* ^m/Y^ animals, or irradiated animals 24 hours after 4 Gy IR were stained for CD4 and CD8 and subjected to surface marker profiling by Flow cytometry. A representative result from one out of two animals is shown. +/Y: wildtype; m/Y mutant. Numbers indicate the percentage of CD4/CD8 double-positive cells in the top-right quadrant. (**B**) Result of AVPI experiment from a representative pair of thymocyte cultures. Thymocytes were cultured overnight after collection and subjected to 10 Gy IR. Cells were collected at various time points after IR and stained with AVPI. Bottom: Percentage of viable (AV^−^, PI^−^), early apoptotic (AV^+^, PI^−^) and late apoptotic (AV^+^, PI^+^) cells from two independent experiments (filled square: *Tspyl2^+/Y^*, open circle: *Tspyl2^m/Y^*, n = 5).

Both cell cycle arrest and apoptosis are protective responses against DNA damage. In the case of thymocytes, more than 95% of cells are resting at the G0/G1 phase before IR and therefore they are not suitable for demonstrating cell cycle arrest. Upon IR, CD4/CD8 double-positive thymocytes undergo apoptosis, and this process is well-characterized to be dependent on the p53 activity, both *in vitro* and *in vivo* [30]. Thymocytes isolated from irradiated mice underwent IR-induced apoptosis, therefore the percentage of CD4/CD8 double-positive cells dropped (Fig. 6A). In wildtype, the percentage of CD4/CD8 double-positive cells dropped from 78.2% ±2.2% to 18.6% ±1.1% (n = 2). *Tspyl2^m^* mutant cells showed a similar reduction from 75.3% ±1.02% to 26.6% ±2.5% after IR (n = 2). To substantiate the *in vivo* findings, mutant and wildtype thymocytes were collected from non-irradiated mice and cultured overnight. After acute exposure to 10 Gy IR, they showed the same rate of cell death as measured by AVPI staining (Fig. 6B). Therefore, our data do not indicate a role of TSPYL2 in apoptosis.

## Discussion

As a novel NAP identified in a number of screens, TSPYL2 is shown to play a role in cell proliferation [6], [11], [12], TGF-β1 signaling pathway [15], [31], [32] and synaptic function [25], [33], [34]. Very little is known about the physiological functions of NAPs and we address this question through gene targeting in mice. This is the first report on mutants amongst the TSPY family. While the loss of TSPYL2 has no deleterious effects in mice we show there is a clear cell cycle defect under the situation of DNA damage. Our results indicate that TSPYL2 is required for cell cycle maintenance in stress condition.

Using MEFs derived from the mutant mice, we are able to reveal the role of TSPYL2 in cell cycle regulation. *Tspyl2* mutant MEFs are defective in G1 cell cycle arrest upon IR. More specifically, initiation of checkpoint response is normal as indicated by proper triggering of the DNA damage response, accumulation of p53 and p21 protein, and reduction in S phase cells within 8 hours after IR. Failure in maintaining the checkpoint arrest in mutant cells is in agreement with the molecular finding of impaired transcriptional activation of *p21*. Upon stressful stimuli such as DNA lesions and survival crisis, transcription of *p21* is activated which serves as the key initiator for cell cycle arrest. As p21 mutant MEFs are also defective in blocking S phase entry after IR but have normal mitotic spindle checkpoint induced by microtubule inhibitor, we attribute the impaired G1 checkpoint in TSPYL2 mutant MEFs mainly to impaired *p21* induction. Other similarities between *Tspyl2* and *p21* knockout mice include the absence of gross developmental abnormalities or spontaneous malignancies. Besides, apoptosis in thymocytes is not affected [35]. Our data suggest that TSPYL2 is a new player in transcription activation of *p21* upon IR.

What causes the specific effect of TSPYL2 on the transcription of *p21* but not several other p53 target genes being tested? In a previous report, reporter activity of the *p21* promoter is significantly activated by cotransfection of TSPYL2 expression plasmid in HeLa cells. This is linked to the increased stability of p53 protein [12]. Our data indicate that in MEFs, p53 stabilization upon IR is normal even without functional TSPYL2. Concerning the role of TSPYL2, it has been proposed that binding of NAPs to nucleosomal DNA facilitates the binding of transcription factors to their DNA binding site [36]. NAPs also strengthen the binding between transcription factor and p300/CBP and this leads to transcription activation [2]. Another possibility is NAPs perform their task of nucleosome displacement after being recruited to the transcription complex [37]. In any case, TSPYL2 is likely to recognize specific partner other than p300/CBP to fine tune the transcription activity of specific p53 target genes.

G1 checkpoint is vital for delaying cell cycle progression upon DNA damage, allowing more time for DNA repair and preventing the replication of mutated template. *Tspyl2* knockout MEFs showed defective cell cycle arrest, but *Tspyl2* mutant animals did not suffer a higher tendency of tumorigenesis when compared with wildtype littermates throughout eighteen months of observation. Furthermore, mutant mice irradiated at 4 Gy did not develop tumors after 6 months (data not shown). Due to the intricate network of processes to control cell proliferation, there is potential compensatory effect by other tumor suppressors. Very often the cell cycle defects are revealed only by introduction of additional mutation in another gene of the same family or working in the same pathway, such as that observed in *p21*, *atm* double mutants and *Chk1*, *Chk2* double mutants [38], [39]. In addition, the genetic background of the mutant animals can affect the tumorigenic observations [40]. We conclude that TSPYL2 is a new player in controlling the cell cycle upon DNA damage.
